# Successful clozapine rechallenge after myopericarditis: a case report

**DOI:** 10.1097/YIC.0000000000000407

**Published:** 2022-05-30

**Authors:** Andrea Boscutti, Guido Cereda, Matteo Lazzaretti, Paolo Enrico, Alessio Fiorentini, Cecilia Prunas, Antonio Callari, Elisa Fontana, Giuseppe Delvecchio, Paolo Brambilla

**Affiliations:** a Department of Pathophysiology and Transplantation, University of Milan and; b Department of Neurosciences and Mental Health, Fondazione IRCCS Ca’ Granda-Ospedale Maggiore Policlinico, Milan, Italy

**Keywords:** clozapine, myocarditis, pericarditis, rechallenge, schizophrenia

## Abstract

Clozapine-induced myocarditis and pericarditis are uncommon adverse effects of clozapine treatment. However, in most cases, they lead to clozapine discontinuation. Here, we describe a case of successful clozapine rechallenge after clozapine-induced myopericarditis. The patient, a 31-year-old male with treatment-resistant schizophrenia (TRS), developed dyspnea on exertion and chest pain on day 19 after the start of clozapine titration. An electrocardiogram (ECG) showed widespread, mild, convex ST interval elevation. While troponin levels were mildly elevated, the echocardiogram was unremarkable. A myopericarditis diagnosis was formulated, and clozapine was stopped, with a progressive resolution of clinical, laboratory and ECG abnormalities. After 6 months, a rechallenge with clozapine was attempted. A very slow titration scheme was adopted, along with close monitoring of clinical, laboratory and ECG parameters. Clozapine target dose was reached without the occurrence of any abnormality. Given the unique role of clozapine in the management of TRS, clozapine rechallenge may be considered after pericarditis, even with troponin levels elevation. Further studies are needed to update current clinical guidelines.

## Introduction

Clozapine is the antipsychotic of choice for treatment-resistant schizophrenia (TRS) (Brambilla *et al.*, 2002; Correll and Howes, 2021). It is commonly associated with mild cardiovascular side effects, including tachycardia, hypotension and electrocardiogram (ECG) abnormalities (Yuen *et al.*, 2018). More severe cardiovascular adverse events, such as clozapine-induced myocarditis (CIM) and pericarditis, are rare, but in most cases lead to clozapine discontinuation.

In the following report, we describe a successful clozapine rechallenge after a case of clozapine-induced pericarditis with myocardial involvement (myopericarditis).

## Case description

A 31-year-old male with schizophrenia was admitted to our inpatient unit for an acute psychotic exacerbation on 5 February 2021. After being diagnosed with TRS based on the most recent guidelines (Howes *et al.*, 2017), the patient was started on clozapine on day 5 after admission. The following titration schedule was adopted: day 1, 25 mg; day 2, 50 mg; day 3, 75 mg; day 6, 100 mg; day 7, 150 mg and day 8, 200 mg. Before starting the patient on clozapine, a baseline screening was performed, including complete blood count (CBC), C-reactive protein (CRP), troponin T (TnT), ECG and echocardiogram. None of the tests showed any significant abnormality. On day 11, the patient developed a cough and sore throat. On day 17, given the worsening of symptoms and increase of CRP (15.04 mg/dL), a chest X-ray was performed, showing accentuated central bronchovascular markings bilaterally, and a right middle lobe consolidation. The patient was started on empirical antibiotic therapy.

**Fig. 1:**
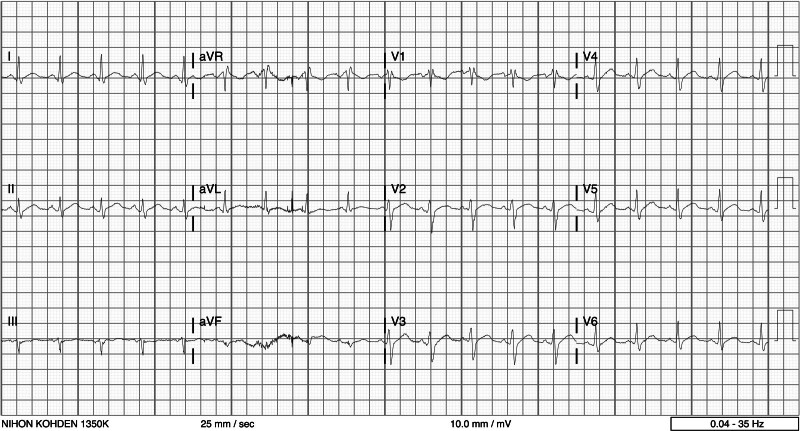
Electrocardiogram showing widespread, mild, convex ST interval elevation, suggesting pericardial involvement.

On day 19, the patient started to complain of dyspnea on exertion and chest pain. Blood tests were performed, showing mild TnT elevation (198 pg/mL) and CRP of 14.33 mg/dL. Compared to baseline findings, a new ECG showed a widespread, mild and convex ST interval elevation (Fig. 1). A new echocardiogram was also performed but showed no abnormalities. Cardiology was consulted and concluded that the clinical picture was consistent with a diagnosis of myopericarditis. Clozapine, at that time up titrated to 300 mg/day, was stopped, and olanzapine was started at 20 mg/day. In the following days, there was a gradual resolution of symptoms, along with a progressive reduction of inflammatory indices and TnT levels, until normalization. On day 27, ECG and echocardiogram were repeated, showing no abnormalities. After discharge from the inpatient unit, the patient was admitted to the high-intensity psychiatric rehabilitation unit of our hospital.

Given the persistence of severe negative symptoms, a trial with brexpiprazole 4 mg/day as an add-on therapy to olanzapine was performed, with unsatisfactory results. Moreover, a subsequent down titration trial of olanzapine at 5 mg/day, with the aim of minimizing the metabolic side effects of the drug, led to an increase in the severity of positive symptoms.

Given the young age of the patient, the unsatisfactory response to previous antipsychotic therapies and the availability of medical and nursing assistance, a clozapine rechallenge was attempted in the high-intensity psychiatric rehabilitation unit of our hospital, 6 months after the first clozapine trial (4 August 2021). Brexpiprazole was stopped before clozapine was restarted. A cross-titration was performed between clozapine and olanzapine. The dose of the latter was gradually decreased with the start of the clozapine rechallenge and finally stopped when the clozapine dose reached 200 mg/day.

A slower titration rate than suggested by guidelines (Taylor and Barnes, 2018) was chosen (Table 1). In addition, a close monitoring protocol was adopted, which included a three times daily measurement of temperature, blood pressure and heart rate. Blood tests (CRP, N-terminal prohormone of brain natriuretic peptide (NT-proBNP), TnT, creatinine, GPT, electrolytes and clozapine plasma level) and ECG were performed at baseline and weekly after clozapine rechallenge. A baseline echocardiogram was also obtained. There was no significant alteration in any of the assessments until a maintenance dose of 350 mg/day was reached after 63 days (Table 1).

**Table 1:** Clozapine titration schedule, results of laboratory tests, and PANSS scores during clozapine rechallenge

Days since start of rechallenge	Clozapine oral dose (mg/day)	CRP (mg/dL)	TnT (pg/mL)	NT-proBNP (pg/mL)	PANSS positive (Kay *et al.*, 1987)	PANSS negative (Kay *et al.*, 1987)	PANSS general (Kay *et al.*, 1987)
0	0	2.89	8.67	25.9	26	40	60
7	37.5	0.45	19	91.4	–	–	–
14	75	5.58	16.8	105	–	–	–
21	125	2.48	11.2	18.7	–	–	–
28	175	NA	11.5	14.8	–	–	–
35	200	0.73	10.3	17.5	–	–	–
42	250	1.29	9.67	54.8	–	–	–
49	300	2.59	12.2	<5	–	–	–
56	325	0.74	11.7	5.74	–	–	–
63	350	0.89	12.5	<5	19	31	48

CRP, C-reactive protein; NT-proBNP, N-terminal prohormone of brain natriuretic peptide; PANSS, Positive and Negative Syndrome Scale (positive, negative and general Psychopathology scales); TnT, Troponin T.

## Discussion

To date, evidence on clozapine rechallenge after the occurrence of CIM and pericarditis is limited to a few reports; for this reason, most recent guidelines do not advise for clozapine rechallenge after pericarditis or CIM (Manu *et al.*, 2018). High success rates of clozapine rechallenge are described after CIM (Rosenfeld *et al.*, 2010; Ronaldson *et al.*, 2012a;). Only three reports of rechallenge after pericarditis can be found, with two of them being successful (Malhotra *et al.*, 2002; Crews *et al.*, 2010), and one reporting a pericardial effusion relapse after clozapine rechallenge (Daly *et al.*, 1992). In a fourth case, pericarditis spontaneously resolved without the discontinuation of the drug (Kay *et al.*, 2002).

While limited and low-quality, this evidence seems to suggest that, in the case of pericarditis, rechallenge could be considered as an option in selected cases, given the unique role of clozapine in the management of TRS. Moreover, recent longitudinal studies indicate that pericarditis carries a good prognosis in the long term. Of note, troponin levels do not appear to influence the risk of complications (Imazio *et al.*, 2013). This may suggest that the occurrence of acute pericarditis with evidence of troponin elevation but without newly developed signs of focal or diffuse impairment of left ventricular function (i.e. myopericarditis) (Adler *et al.*, 2015) should probably not considered as a negative prognostic factor for rechallenge.

Because the risk of relapse has been associated with the speed of drug titration (Ronaldson *et al.*, 2012b), adopting a very slow titration schedule may be preferable, as already described for CIM (Shivakumar *et al.*, 2020). In addition, a three times daily monitoring of vital signs (temperature, blood pressure and heart rate) could facilitate early detection of relapse, together with weekly monitoring of laboratory parameters (CBC, CRP, NT-proBNP and TnT) and ECG.

## Acknowledgements

Authors agree to make data and materials presented in their article available upon reasonable request.

Written informed consent was obtained from the individual for the publication of any potentially identifiable images or data included in this article.

Conceptualization: A.B., G.C. and M.L.; writing – original draft preparation: A.B., G.C. and M.L.; writing – review and editing: A.B., G.C., M.L., P.E., A.F., C.P., A.C., E.F., G.D. and P.B.; supervision: P.B.

### Conflicts of interest

There are no conflicts of interest.
